# Understanding the Role of Gui-Zhi-Fu-Ling-Capsules (Chinese Medicine) for Treatment of Endometriosis in the Rat Model: Using NMR Based Metabolomics

**DOI:** 10.1155/2018/9864963

**Published:** 2018-12-19

**Authors:** Jue Zhou, Zhi-Ming Ding, Paul J. Hardiman

**Affiliations:** ^1^College of Food Science and Biotechnology, Zhejiang Gongshang University, Hangzhou 310018, China; ^2^Institute for Women's Health, Medical School, University College London, London NW3 2PF, UK; ^3^Women's Hospital, School of Medicine, Zhejiang University, Hangzhou 310006, China

## Abstract

The objective of this study is to identify the changes of metabolites in the rat endometriosis models treated with* Gui-Zhi-Fu-Ling-capsules *(GZFLC), a classic Chinese medicinal formula, and to explore the effects of GZFLC on the serum levels of transforming growth factor-*β*1 (TGF-*β*1) and the mRNA expression levels of vascular endothelial growth factor (VEGF) and glucose transporter 4 (GLUT-4) in the endometriotic tissues. Forty female Wistar rats were randomly divided into the sham-operation group (Normal group), Model group, Danazol group, and GZFLC group. The serum levels of TGF-*β*1 were measured using enzyme-linked immune-sorbent assay (ELISA). The mRNA expression levels of VEGF and GLUT-4 in the endometriotic tissue of the rat endometriosis models were measured using real-time quantitative PCR. The metabolites in urine were detected by ^1^H NMR method. Eight identified metabolites of the NMR resonance were involved in the glycolysis metabolism. Among the 8 metabolites, Lactate, Acetate, TMA, and Formate were downregulated with GZFLC. Citrate, TMAO, Taurine, and Hippurate were unregulated with GZFLC. The serum levels of TGF-*β*1 in the Danazol and GZFLC groups were significantly higher than those of Normal group and significantly lower than the Model group. GZFLC treatment significantly decreased the GLUT-4 and VEGF mRNA expression levels in the endometriotic tissues of the endometriosis rats (*P *< 0.05). GZFLC significantly decreased the GLUT-4 mRNA expression levels in rats of GZFLC group compared with Danazol group. It is through regulating the metabolites changes of glycolysis or gluconeogenesis that GZFLC significantly affected the expression levels of TGF-*β*1, GLUT-4, and VEGF of the model rats with endometriosis.

## 1. Introduction

Endometriosis is the most common gynecological disease, which is characterized by the growth of endometrial tissue at ectopic locations [1]. It is a chronic condition characterized by the presence of endometrial tissue outside the uterine cavity, most commonly on the pelvic peritoneum and ovaries [2, 3]. Endometriosis can result in substantial morbidity, including pelvic pain, multiple operations, and infertility [3]. Endometrial tissue is characterized by an exaggerated inflammatory environment around ectopic tissues [4, 5]. Endometriosis affects approximately 10% of women of reproductive age [3, 6]. About 30% of the patients are asymptomatic and the incidence of infertility among women suffering from endometriosis ranges from 30%-40% [6]. Increased peritoneal fluids concentrations of cytokines that lead to the migration, proliferation, and activation of macrophages have been reported in patients with endometriosis [7, 8]. Surgery provides relief to women in pain, but symptoms recur in 75% of cases within 2 years [9].

Although surgical and hormonal treatment are applied as the common interventions, the unpleasant side effects and high rates of relapse limited the range of these interventions [10, 11]. Some traditional Chinese medicinal herbs, such as Bupleurum (Chinese name, Chai hu) and Chinese angelica (Chinese name, Dang Gui), have significant curative effects in treating endometriosis [12, 13]. For traditional Chinese medicinal formulas, Gui-Zhi-Fu-Ling formula, Dang-Gui-Shao-Yao-San, Jia-Wei-Xiao-Yao-San are the most frequently prescribed Chinese herbal products (CHPs) to treat endometriosis [14].

Gui-Zhi-Fu-Ling-Wan (GZFLW) is a classic Chinese medicinal formula, which was firstly described in Essential Prescriptions from the Golden Cabinet (Jingui Yaolue) by an ancient Chinese doctor, Zhong-Jing Zhang, Han Dynasty (300 A.D.) [15–17]. GZFLW is one of the top ten most frequently prescribed CHPs used to relieve endometriosis-related symptoms [14]. GZFLW consists of five herbs: Ramulus Cinnamomi, Poria, Semen Persicae, Radix Paeoniae Rubra or Radix Paeoniae Alba, and Cortex Moutan. The traditional effects of GZFLW are invigorating blood, dissolving stasis, and resolving masses [15] and it has sedative and anti-inflammatory effects on endometriosis-related symptoms [14].* Gui-Zhi-Fu-Ling Capsules *(GZFLC) originate from the classic Chinese medicinal formula, GZFLW, which is more convenient to be taken and easily accepted by majority of the patients.

The metabolic disorder has been found to play an important role in the development of endometriosis, and rats' urinary metabolomes have revealed the potential roles of CHPs in management of metabolic disorder [18, 19]. Metabonomics is a scientific discipline that can be used to study changes in the metabolite ensembles associated with disease pathophysiology [20]. 1H-NMR based metabolomics, a rapid and noninvasive approach, to identify metabolic changes associated with endometriosis in urine samples, is useful to get a better understanding of the pathogenesis of endometriosis, thus providing support to the noninvasive diagnosis of this pathology [21]. Few studies have been conducted on the mechanism and the functions of GZFLC in relation to the metabolic changes in pelvic endometriosis. Therefore, the present study was then designated to identify the changes of metabolites in the rat endometriosis models treated with GZFLC and to explore the effects of GZFLC on the levels of TGF-*β*1 and the mRNA expression levels of vascular endothelial growth factor (VEGF) in the endometriotic tissues.

## 2. Materials and Methods

### 2.1. Materials

GZFLC is a Chinese formula, composed of five Chinese medicinal plants, Cinnamomum cassia Blume (Cinnamomi cortex), Paeonia lactiflora Pallas (Paeoniae radix), Paeonia suffruticosa Andrews (Moutan cortex), Prunus persica Batsch (Persicae semen), and Poria cocos Wolf (Hoelen). The capsules of GZFLC were purchased from Beijing Tong Ren Tang Limited, China. The voucher specimens of GZFLC were deposited in the laboratory of College of Food Science and Biotechnology, Zhejiang Gongshang University, Hangzhou, China. The dried powder of the capsules was suspended in 0.5% sodium carboxymethyl cellulose (CMC Na) before being administered orally with the dose of 40mg/kg body weight to rats. The oral dosage for rats was calculated according to the patients dosages of GZFLC used 2.7g per day. Danazol was provided by Lianhua Medicine Company (Jiangsu, China).

### 2.2. Establishment of the Model Rats with Endometriosis

40 female Wistar rats with body weight of 160 ±20 g were purchased from the Laboratory Animal Center of Zhejiang Chinese Medical University (Hangzhou, China). The experimental protocol was approved by Zhejiang Gongshang University. The animal's welfare was monitored according to the Guide for the Care and Use of Laboratory Animal of the National Institute of Health (Publication No. 80–23, revised 1996). We attempt to minimize suffering of animals and reduce the numbers of animal in the study. Among the 40 rats, 10 rats were randomly taken as the Normal control group. The method of establishing the model rats with endometriosis was based on our previous studies [22–25]. Four weeks after the endometriosis model were made, the 30 model rats were randomly divided into three groups (n=10 in each group) including model control group (Model group), GZFLC group, and Danazol group. After all the treatment ended, the 40 rats were all sacrificed.

### 2.3. Group and Administration

The 40 rats were divided into four groups: GZFLC group (the rats were orally administrated with the extracts of GZFLC at 40 mg/kg once daily for 30 consecutive days), Danazol group (the rats were orally administrated with Danazol at 36 mg/kg once daily for 30 consecutive days), Model Control group (Model group, the rats were orally administrated with saline at 8ml/kg once daily for 30 consecutive days), and Normal group (the rats were orally administrated with saline at 8ml/kg once daily for 30 consecutive days). After all the treatment ended, the rats were sacrificed and the samples of endometriotic tissue and the serum were taken. A total of 0.1g endometriotic tissue was collected and stored in TRIzol reagent (Invitrogen, Carlsbad, CA, USA) and stored frozen at −80°C until being used for further evaluation. The endometriotic tissue was homogenized in the tissue homogenizer. Then CCL_4_ was added and centrifuged at 12,000 rpm for 7 min at 4°C. The supernatants were collected and stored frozen at −80°C until being used for further evaluation. After GZFLC o.a. for 30 consecutive days, at day 31, urine samples were collected in metabolism cages overnight from all groups and then frozen at −20°C and stored until analysis. The levels of Lactate in urine were detected by NMR. The serum levels of transforming growth factor-*β*1 (TGF-*β*1) were detected with ELISA. The mRNA expression levels of vascular endothelial growth factor (VEGF) and glucose transporter 4 (GLUT-4) were detected with qPCR.

### 2.4. Rat Urine Sample Preparation

Urine samples were collected in metabolism cages overnight from all groups. Then, urine samples were frozen at −20°C and stored until analysis. A 1ml aliquot of each of the urine samples was taken for ^1^H-NMR spectroscopy. To reduce pH induced chemical shift variability in the urine samples, 200*µ*L of phosphate buffer (0.2 mol/L KH_2_PO_4_, 0.8 mol/L KH_2_PO_4_) were added to 500*µ*L urine then centrifuged at 8000x g for 5 min. 10*µ*L of sodium trimethylsilyl propionate (TSP) and 50*µ*L of deuterium oxide (D_2_O) were added to 550*µ*L of the supernatant. TSP was used as a chemical shift reference. 10% D_2_O was used as a lock solvent for high resolution for NMR spectrum. Push solvent (1g NaN_3_ in 1000 ml dH_2_O) was used for NMR automation [26].

### 2.5. ^1^*H* NMR Analysis of Metabolites in Urine

All the ^1^H NMR spectra were acquired from Bruker 600 MHz spectrometer (Bruker BioSpin, Germany). Free induction decay was zero filled to 64 K points then multiplied with an exponential function to 1.0 Hz line-broadening factor before Fourier transformation. The ^1^H NMR spectra between *δ* 0.5 and 10.0 were segmented into consecutive nonoverlapping regions of *δ* 0.002 chemical shift bins. We chose the bin size of *δ* 0.002 as the primary chemical shift width for the identification of metabolites and integrated the spectral area in each bin, which can avoid the encompassment of several peaks and have clear peak multiplicities. The water resonance of *δ* 4.50–5.23 and urea resonance of *δ* 5.46–6.25 were excluded to eliminate baseline effects. We use the division of integrated segment with the total area of the spectrum to compensate the differences of concentration [27]. The peak area of each bin was calculated.

### 2.6. Multivariate Statistical Techniques of ^1^*H* NMR Data

Multivariate analysis of ^1^H NMR data was analyzed with SIMCA-P+ (version 11.5.0.0; Umetrics AB, Umeå, Sweden). Mean centered integral values were used for the principal component analysis (PCA), partial least square discriminant analysis (PLS-DA), and OPLS-DA analysis. Unsupervised PCA was used for the data of initial visualization. PLS-DA was used for the further trends of the data. The quality of PLS-DA models was analyzed using R^2^ and Q^2^, where R^2^ is the estimate of goodness-of-fit of model to the data, while Q^2^ is the estimate of goodness of prediction.

### 2.7. Determination of TGF-*β*1 in Serum

2 ml of blood was drown in polypropylene tubes and was allowed to clot for 30 minutes before centrifugation. The serum was obtained by centrifugation for 10 minutes at approximately 1000 g. The samples were stored at - 70°C before the measurements (not longer than 3 months). Serum TGF-*β*1 concentrations were measured using commercially available ELISA kit (Neobioscience, Shen-zhen, China).

### 2.8. Detection of GLUT-4 and VEGF mRNA Expression Levels in Endometriotic Uterus Tissue

A total of 0.1g endometriotic tissue was collected and stored in TRIzol reagent (Invitrogen, Carlsbad, CA, USA). The tissue was homogenized in the tissue homogenizer (30 s at 4.0 m/s). The relative expression was determined by quantitative real-time PCR (Q-RT PCR) in an ABI Step-one Sequence Detection System (Applied Biosystems, Forrest City, CA, USA). The total RNA was isolated with RNAiso™ reagent (Takara Biotechnology, Dalian, China) according to the instructions of the manufacturer. The purity and concentration of the RNAs were determined by OD260/280 readings with a NanoDrop® ND-100 spectrophotometer (Thermo Fisher Scientific Inc., Waltham, MA, USA). The ratio of absorbance at 260 nm and 280 nm is used to assess the purity of DNA and RNA. A ratio of 1.8-2.0 is generally accepted as pure for RNA. The cDNA was prepared from 500 ng total RNA by reverse transcription (RT) with the PrimeScript™ RT Reagent kit (Perfect Real Time; Takara Biotechnology). The cDNA samples were then diluted in DNase- and RNase-free water at a proportion of 1:3 prior to further analysis. For the mRNA quantification, the normalized Ct of each gene was compared with the endometriotic tissue of control group mRNA. The rat GLUT-4 and VEGF gene-specific primers were provided by Sangon Biological Engineering Technology (Shanghai, China). PCR reactions were performed in a final volume of 25 *μ*l containing 50 ng of templates complementary DNA (cDNA) and 2.5 *μ*l of 10× PCR buffer real-time polymerase chain reaction (PCR) amplifications for GLUT-4 and VEGF were conducted using One-Step SYBR PrimeScript RT-PCR Kit according to the manufacturer's instructions under the following conditions: 95°C for 5min, followed by 40 cycles of 95°C for 5s and 60°C for 10s. Polymerase chain reaction products of *β*-actin primer gene were used as an internal standard. Specificity of amplification was confirmed by melt curve analysis. After normalization against *β*-actin mRNA, the comparative threshold (CT) method (i.e., ΔΔCT method) was used to quantify the relative expression levels of the samples. Fold changes were calculated using the equation 2-ΔΔCT. Q-RT PCR experiments were repeated three times independently. Data are represented as the mean ±SD.

### 2.9. Statistical Analysis

Processing of the NMR spectra was performed by comparing spectral overlays using Topspin 3.0 (Bruker Biospin). The shifting signals were aligned in MATLAB 7.13 (MathWorks, Inc., Natick, MA, USA). Regular and dynamic bins were applied. Dynamic bins were determined with MATLAB for the Dynamic Adaptive Binning (DAB) Algorithm (Anderson et al., 2011). The statistical analysis was conducted using the Simca-P+ software (Umetrics, Umeå, Sweden). Data were analyzed using the Statistical Package for Social Sciences (SPSS 22.0 for Windows). The comparisons of different groups were performed with one-way analysis of variance (ANOVA) and multiple comparison tests with Bonferroni correction procedure. For all the hypothesis tests, significance level was set at* P*=0.05 and two-tailed tests were used.

## 3. Results

### 3.1. ^1^*H* NMR Metabolomic Analysis of Urine Samples

**Figure 1:**
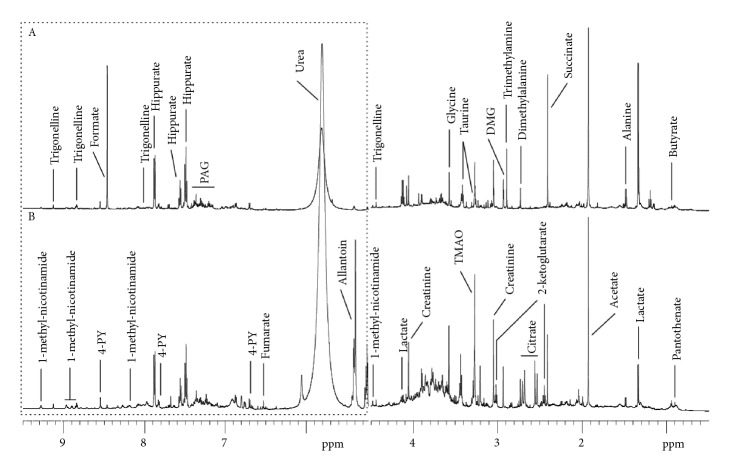
600 MHz ^1^H-nuclear magnetic resonance spectra (*δ* 0.5-4.7, 5.0-9.6) of urine samples obtained from (A) control group, (B) GZFLW group. Keys: DMG: dimethylglycine; TMAO: Trimethylamine N-oxide; PAG: Phenylacetylglycine; 4-PY: N-methyl-4-pyridone-5-carboxamide.

The urine samples were subjected to ^1^H NMR analysis to investigate the metabolic changes in the urine caused by GZFLC treatment. All the rats were divided randomly into two groups for NMR analysis, with 8 rats in each group: A, control group (endometriosis model rats without any treatment; B, GZFLC group, endometriosis model rats with GZFLC treatment. Representative 600MHz 1H NMR spectra (*δ* 0.5-4.7, 5.0-9.6) from group A and group B were shown in Figure 1. Data obtained from the ^1^H NMR spectra were analyzed. The data were subsequently analyzed using multivariate statistics (PCA, PLS-DA, and OPLS-DA). The metabolites were identified based on data obtained from database of Human Metabolome Database (HMDB).

**Figure 2:**
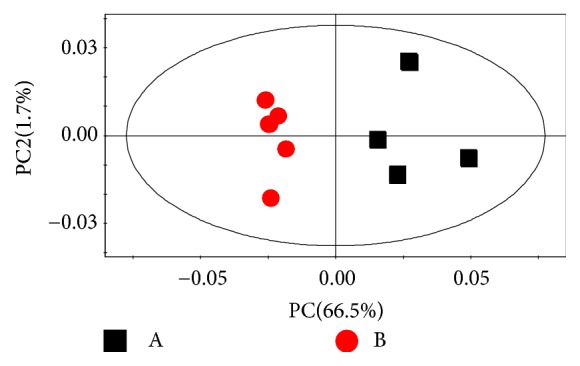
Principal component analysis (PCA) scores plot PC vs. PC2 obtained from ^1^H NMR spectra of urine samples from two groups, (A) control group, (B) GZFLW group. The ellipse represents 95% confidence region of the model based on HotellingT2. R^2^X=82.2%, Q^2^=0.54.

The PCA scores plots of ^1^H NMR data in Figure 2 give an overview of the profiles for the respective treatments. The PCA and PLS-DA score plots were visualized with the first principal component (PC) and the second principal component (PC2). Based on R^2^X=82.2%, Q^2^=0.54 (Figure 2), the mathematical model is effective. The clustering phenomenon between the two groups is obvious. There is significant difference between the control group and the GZFLC group.

### 3.2. Discrimination between the Control and GZFLC Groups Using ^1^*H* NMR

**Figure 3:**
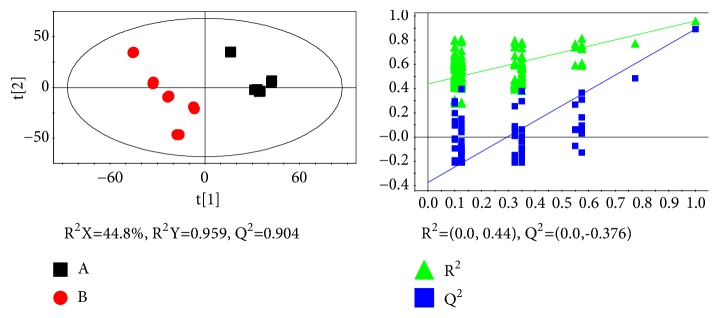
Scores plot (top) and validation plot (bottom) of partial least squares discriminant analysis (PLS-DA) models from two groups. (A) Control group(■) and (B) GZFLW group(●).

The PLS-DA was based on a unit variance scaling strategy. A 10-fold cross-validation was used to validate the PLS-DA model, which was performed to evaluate the quality of the model by parameters R^2^X, R^2^Y, and Q2. R^2^X represents the total variation in X and describes the optimization of the analytical model. R^2^Y represents the variation in the response variable Y. Q^2^ represents the predictive ability of the model and the authenticity of the predicted results. On the scores plot, each point represented an individual sample. The center and the margin of each ellipse indicate mean and standard deviation, respectively. A permutation test from the verification plots was performed to validate the degree of overfitting for the PLS-DA model. The correlation coefficient between the original Y and the permutated Y was plotted against the cumulative R^2^ and Q^2^; a regression line was calculated with the R^2^ and Q^2^ intercept limits. These tests compared the goodness-of-fit of the original model with the goodness-of-fit of several models based on data in which the order of the Y observations was randomly permutated while the X matrix remained constant. In the verification plots, the Y-axis represents R^2^ (green triangle) and Q^2^ (blue box) and the X-axis designates the Pearson correlation coefficient between the original and permutated rank-ripeness. When the Q^2^ regression line had a negative intercept and R^2^ values on the left were lower than the original points on the right, the validation plots of the PLS-DA model were effective. The scores plots and validation plots of PLS-DA are shown in Figure 3. Based on the PLS-DA models in Figure 3, group A and B were discriminated with an R^2^X of 0.448, an R^2^Y of 0.959, and a Q^2^ of 0.904. The results showed that the original PLS-DA models were effective. As shown in Figure 3, there is significant difference between the rats of the control group and the GZFLC group.

**Figure 4:**
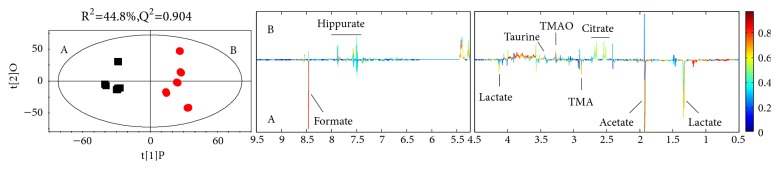
OPLS-DA scatter plots derived from 1H NMR spectra of the two groups. Notes: (A) control group(■), (B) GZFLW group(●). R^2^X=0.448 and Q^2^=0.904. The vertical axis of the validation plots represented the R^2^ and Q^2^ values, and the horizontal axis represented the correlation coefficients.

OPLS-DA plots were visualized with the first principal component (t[1]) and the orthogonal component (t[2]). The parameters Q^2^ and R^2^X were computed to test the validity of the model against overfitting, where R^2^X is the total variation explained in the data and Q^2^ is the cross-validated explained variation with increasing reliability as Q^2^ approaches 1. The sixfold cross-validation method and permutation test for 500 times with the first component were carried out to measure the robustness of the model, where if Q^2^ (max) obtained from permutation test is less than or equal to Q^2^ (cum) obtained from OPLS-DA, the established OPLS-DA model is robust. Based on the OPLS-DA models, the rats in the control and GZFLC group were discriminated with R^2^X = 44.8% and Q^2^ = 0.904. In OPLS-DA scores plots, significant biochemical differences were also observed between the two groups (Figure 4).

By assessing the coefficient plots of OPLS-DA in Figure 4, the main metabolites contributing to the separation of the two groups were shown in Table 1. Among the 8 metabolites, Lactate, Acetate, TMA, and Formate were downregulated with GZFLC. Citrate, TMAO, Taurine, and Hippurate were upregulated with GZFLC. At the end of the study, the two groups were subjected to NMR metabolomic studies. 8 metabolites were identified by fully mapping of chemical shifts, coupling patterns, and coupling constants to previously reported data. In Table 1, discriminating metabolites (with respect to the control group) of the group treated with GZFLC were presented. Eight identified metabolites of the NMR resonance, including Lactate, Acetate, Citrate, TMA (Trimethylamine N), TMAO (Trimethylamine N-oxide), Taurine, Hippurate, and Formate are involved in glycolysis and other metabolisms.

#### 3.2.1. Identification of Predicative Metabolite Pathways

**Figure 5:**
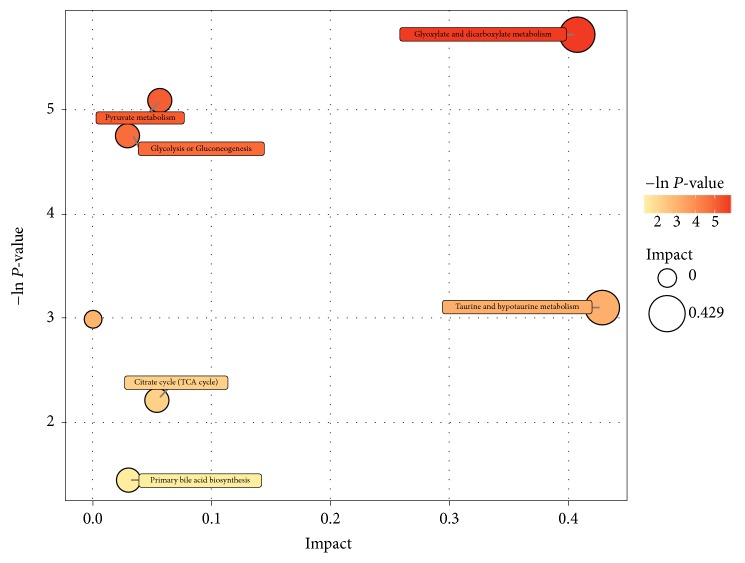
Metabolite pathway mapping of the involved metabolites. The predicative pathway analysis was performed with Simca-P+ software. The results are presented graphically as a bubble plot. The more Darker-color and bigger area of the bubbles represents the more significant metabolite changes in the corresponding pathway.

To determine metabolic pathways which were affected by GZFLC, the metabolites Lactate, Acetate, Citrate, TMA (Trimethylamine N), TMAO (Trimethylamine N-oxide), Taurine, Hippurate, and Formate (Table 1) were mapped with Simca-P+ software. The metabolic pathways were presented graphically as a bubble plot in Figure 5. In Table 2, the pathways and functional analysis in the bubble plot are summarized: (A) Glyoxylate and dicarboxylate metabolism; (B) Pyruvate metabolism; (C) Glycolysis or Gluconeogenesis; (D) Taurine and hypotaurine metabolism; (E) Methane metabolism; (F) Citrate cycle (TCA cycle); and (G) Primary bile acid biosynthesis. The top three pathways and functional metabolites are (A) Glyoxylate and dicarboxylate metabolism; (B) Pyruvate metabolism; and (C) Glycolysis or Gluconeogenesis. Based on the KEGG databases and the pathways and functional analysis of metabolites, glycolysis or gluconeogenesis has the most total numbers of compounds in the pathway.

#### 3.2.2. The Serum Levels of TGF-*β*1

**Figure 6:**
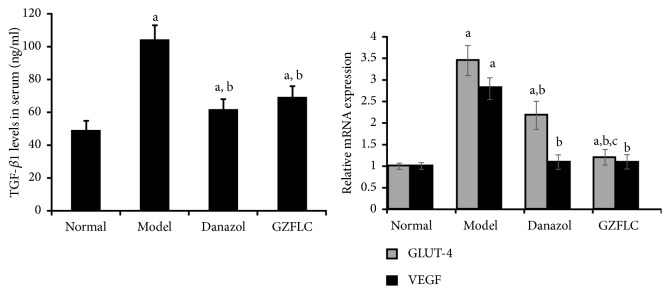
(Left) The serum level of transforming growth factor-beta 1 (TGF-*β*1). (Right) Glucose transporter (GLUT) and vascular endothelial growth factor (VEGF) mRNA expression levels in the endometriotic tissues. Data were shown as mean ± SD (N=10 in each group). The significant difference was set at ^a^*P* < 0.05, compared with the Normal group; ^b^*P*<0.05, compared with the Model group; and ^c^*P*<0.05, compared with the Danazol group.

As shown in Figure 6, the serum levels of TGF-*β*1 in Danazol group and GZFLC group were significantly higher than those of Normal group (*P*<0.05) and significantly lower than the Model group (*P*<0.05). No marked difference existed between the GZFLC and Danazol groups (*P*>0.05).

#### 3.2.3. GLUT-4 and VEGF Gene Expression Levels in the Endometriotic Tissues

For the GLUT-4 mRNA expression levels, it was significantly higher in the Danazol and the Model groups compared with the Normal group (*P*<0.05) (Figure 5). For the GLUT-4 and VEGF mRNA expression levels, there were no significant differences between the GZFLC group and the Normal group. Compared with the Model group, the GLUT-4 and VEGF mRNA expression levels in the endometriotic tissue were significantly lower in the other groups (*P*<0.05). The Model group displayed the highest GLUT-4 and VEGF mRNA expression levels in the endometriotic tissues among all the groups. The GZFLC treatment significantly decreased the GLUT-4 and VEGF mRNA expression levels in the endometriotic tissues of the endometriosis rats (*P*<0.05). GZFLC significantly decreased the GLUT-4 mRNA expression levels in the rats of GZFLC group compared with the Danazol group (*P*<0.05).

## 4. Discussion

The present study focuses on the use of proton nuclear magnetic resonance spectroscopy based targeted metabolite profiling approach to explore metabolites changes expression for the rats with endometriosis treated with Chinese formula GZFLC. Furthermore, association of glycolysis with the metabolite ensembles was also investigated. Xia Liu et al. found Herba Rhodiolae, a different traditional Chinese medicine, has similar infect on the glycolysis metabolisms [28]. In the present study, we identified eight metabolites of the NMR resonance involved in the glycolysis and other metabolisms, which were treated with different medicine* Gui-Zhi-Fu-Ling-capsules *(GZFLC). Among the 8 metabolites, Lactate, Acetate, TMA, and Formate were downregulated with GZFLC. Citrate, TMAO, Taurine, and Hippurate were unregulated with GZFLC. Based on the KEGG databases and the pathways and functional analysis of metabolites, glycolysis or gluconeogenesis has the most total numbers of compounds in the pathway.

Glycolysis is an energy-producing mechanism that occurs in almost all cells and requires an adequate uptake of glucose mediated by glucose transporter (GLUT) proteins [29]. Increased glucose metabolism and defects in the mitochondrial respiratory system are suggested to be the possible sources of excessive reactive oxygen species generation in endometriosis [20]. Endometriotic lesions induced glycolysis, but limited metabolites related researches have been focused on this. Little is known about GLUT proteins expression either in the endometrium or the endometriotic lesions.

Via glycolysis pathway, TGF-*β* induces the metabolic conversion of glucose to Lactate, a process referred to as the “Warburg effect” [30]. Overproduction of Lactate increases cell invasion, angiogenesis, and immune suppression, all crucial steps in the development known regulators of endometriosis [31]. TGF-*β* is believed to play a major role in the etiology of peritoneal endometriosis [9]. Also, TGF-*β*1 stimulated glucose transport and induces changes in the metabolic phenotype of peritoneal mesothelial cells from women with endometriosis.

Adequate glucose uptake and metabolism are essential for the proper differentiation of the uterine endometrium toward a receptive state capable of supporting embryo implantation. The inducible nature of GLUT-4 and its limited cellular expression may make GLUT-4 an attractive target for non-hormone-based treatments of endometriosis [29]. TGF-beta and VEGF are released by inflammatory process cells which may serve as the source of wide spectrum of inflammatory mediators and growth factors [32].

In the proton NMR study, based on the KEGG databases and the pathways and functional analysis of metabolites using, glycolysis or gluconeogenesis has the most total numbers of compounds in the pathway. At the meanwhile, we found that the serum levels of TGF-*β*1 in the Danazol and GZFLC groups were significantly higher than those of Normal group and significantly lower than the Model group. GZFLC treatment significantly decreased the GLUT-4 and VEGF mRNA expression levels in the endometriotic tissues of the endometriosis rats. Compared with Danazol group, GZFLC significantly decreased the GLUT-4 mRNA expression levels in rats of GZFLC group.

To conclude, GZFLC significantly affected the expression levels of TGF-*β*1, GLUT-4, and VEGF of the model rats with endometriosis, accompanied by regulating the metabolites changes of glycolysis. We hypothesized that GZFLC regulates endometriosis by effect on glycolysis or gluconeogenesis. However, more studies should be conducted to explore the underling molecular and metabolism mechanism further.

## Figures and Tables

**Table 1:** Discriminating metabolites (with respect to the control group) of the treated group with GZFLC.

**No.**	**Metabolites**	**Chemical shift**	**Correlation Coefficients **	**HMDB**	**PubChem**	**KEGG**
1	Lactate	1.33	-0.676	HMDB0000190	107689	C00186
2	Acetate	1.91	-0.967	HMDB0000042	176	C00033
3	Citrate	2.55	+0.675	HMDB0000094	311	C00158
4	TMA (Trimethylamine N)	2.88	-0.745	HMDB0000906	1146	C00565
5	TMAO (Trimethylamine N-oxide)	3.27	+0.737	HMDB0000925	1145	C01104
6	Taurine	3.42	+0.796	HMDB0000251	1123	C00245
7	Hippurate	7.56	+0.668	HMDB0000714	464	C01586
8	Formate	8.46	-0.863	HMDB0000142	284	C00058

**Table 2:** Pathways and functional analysis of metabolites.

	**Pathway**	**Total** ^**a**^	**Hits** ^**b**^	**Raw p** ^**c**^	**-ln(p)**	**Holm adjust**	**FDR**	**Impact** ^**d**^
A	Glyoxylate and dicarboxylate metabolism	16	2	0.003287	5.7179	0.26623	0.23354	0.40741
B	Pyruvate metabolism	22	2	0.006219	5.0801	0.49752	0.23354	0.05583
C	Glycolysis or Gluconeogenesis	26	2	0.00865	4.7502	0.68333	0.23354	0.02862
D	Taurine and hypotaurine metabolism	8	1	0.044858	3.1043	1	0.81549	0.42857
E	Methane metabolism	9	1	0.050339	2.989	1	0.81549	0
F	Citrate cycle (TCA cycle)	20	1	0.10884	2.2178	1	1	0.05356
G	Primary bile acid biosynthesis	46	1	0.23476	1.4492	1	1	0.02976

The predicative pathways are (A) Glyoxylate and dicarboxylate metabolism; (B) Pyruvate metabolism; (C) Glycolysis or Gluconeogenesis; (D) Taurine and hypotaurine metabolism; (E) Methane metabolism; (F) Citrate cycle (TCA cycle); and (G) Primary bile acid biosynthesis. Based on the pathways and functional analysis of metabolites, Glycolysis or Gluconeogenesis has the most total number of compounds in the pathway based on the KEGG databases.

^a^The total number of compounds in the pathway based on the KEGG databases.

^b^The actual matched names and numbers of the predicted metabolites.

^c^The P value calculated from the analysis.

^d^The pathway impact value calculated.

## Data Availability

The data used to support the findings of this study are available from the corresponding author upon request.
